# Small extracellular vesicles from mesenchymal stem cells: A potential Weapon for chronic non-healing wound treatment

**DOI:** 10.3389/fbioe.2022.1083459

**Published:** 2023-01-10

**Authors:** Qian Wei, Xi Liu, Jian-Long Su, Ya-Xi Wang, Zi-Qiang Chu, Kui Ma, Qi-Lin Huang, Hai-Hong Li, Xiao-Bing Fu, Cui-Ping Zhang

**Affiliations:** ^1^ Research Center for Tissue Repair and Regeneration Affiliated to the Medical Innovation Research Division and the 4th Medical Center of Chinese, PLA General Hospital, Beijing, China; ^2^ Tissue Repair and Regeneration, Chinese Academy of Medical Sciences, Research Unit of Trauma Care, Beijing, China; ^3^ PLA Key Laboratory of Tissue Repair and Regenerative Medicine and Beijing Key Research Laboratory of Skin Injury, Repair and Regeneration, Beijing, China; ^4^ Department of Wound Repair, Institute of Wound Repair and Regeneration Medicine, Southern University of Science and Technology Hospital, Southern University of Science and Technology School of Medicine, Shenzhen, China

**Keywords:** small extracellular vesicles, chronic non-healing wounds, cell dysfunction, mesenchymal stem cells, regenerative medicine

## Abstract

Chronic non-healing wounds have posed a severe threat to patients mentally and physically. Behavior dysregulation of remaining cells at wound sites is recognized as the chief culprit to destroy healing process and hinders wound healing. Therefore, regulating and restoring normal cellular behavior is the core of chronic non-healing wound treatment. In recent years, the therapy with mesenchymal stem cells (MSCs) has become a promising option for chronic wound healing and the efficacy has increasingly been attributed to their exocrine functions. Small extracellular vesicles derived from MSCs (MSC-sEVs) are reported to benefit almost all stages of wound healing by regulating the cellular behavior to participate in the process of inflammatory response, angiogenesis, re-epithelization, and scarless healing. Here, we describe the characteristics of MSC-sEVs and discuss their therapeutic potential in chronic wound treatment. Additionally, we also provide an overview of the application avenues of MSC-sEVs in wound treatment. Finally, we summarize strategies for large-scale production and engineering of MSC-sEVs. This review may possibly provide meaningful guidance for chronic wound treatment with MSC-sEVs.

## 1 Introduction

**FIGURE 1:**
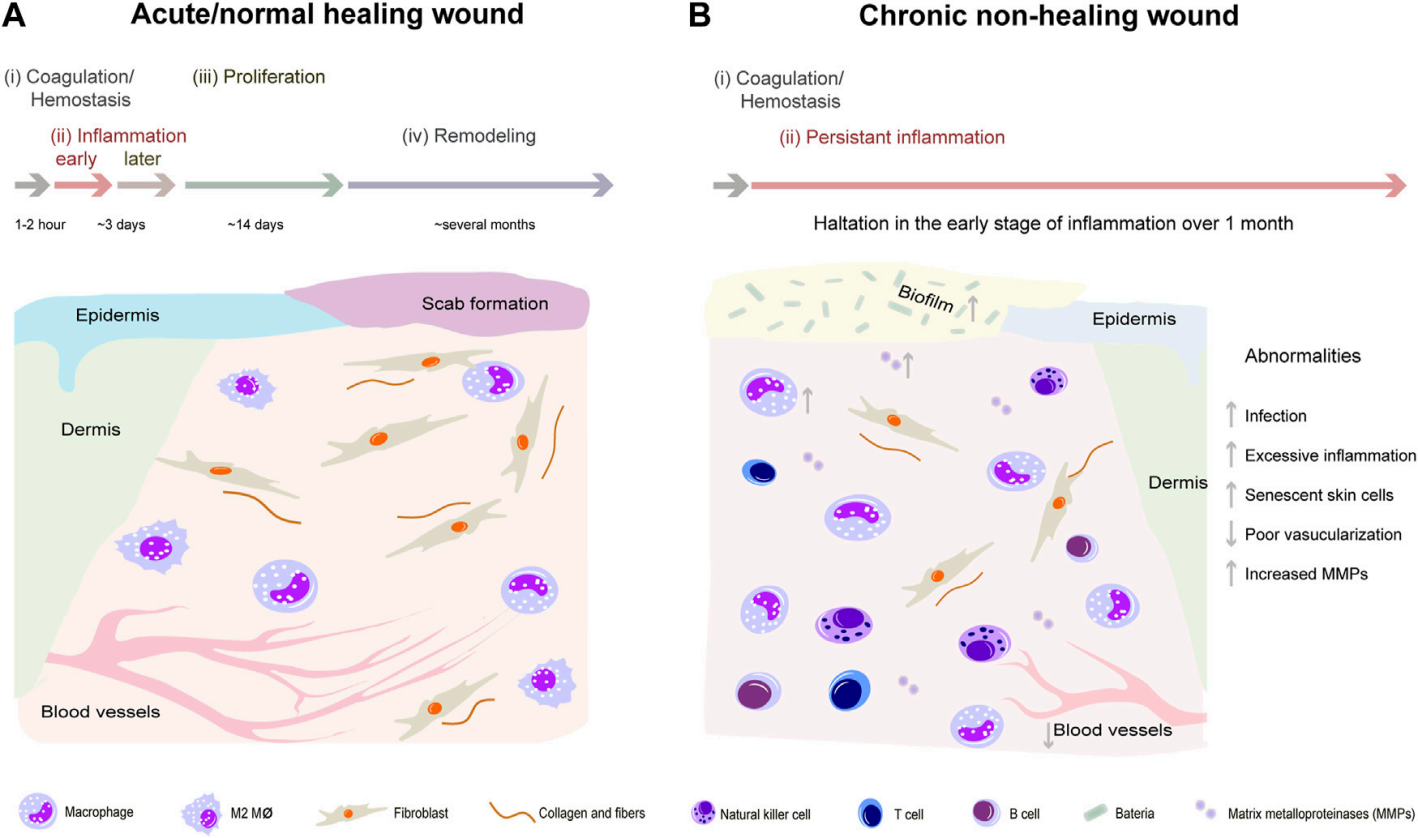
Comparison of representative pathophysiological processes between normal healing wounds and chronic non-healing wounds. Normal wound healing process can be divided into four distinct and overlapping phases: hemostasis, inflammation, proliferation, and remodeling **(A)**. However, chronic wounds usually halt at inflammation phase and are difficult to heal because of excessive inflammation, senescent skin cells, poor vascularization, and increased matrix metalloproteinases **(B)**.

Skin wound healing is a highly complex process participated by many kinds of cells including inflammatory cells, vascular endothelial cells, fibroblasts, epidermal cells, etc. This process can be divided into four distinct and overlapping phases: hemostasis, inflammation, proliferation, and remodeling (Rodrigues et al., 2019). Chronic wounds usually arise owning to halt at one or more points in above phases (Figure 1). With the advent of the global aging society, the number of patients with chronic wounds is increasing, which represent an economic burden worldwide and a heavy burden to patients. For example, in the United States, diabetic foot ulcers, one representative type of chronic non-healing wounds, brought about 130,000 lower-limb amputations in 2016 (Services UDoHaH, 2020). In China, the average hospitalization duration was reported to be 31 days with a medical cost of ¥17,182 (Jiang et al., 2011). Development of effective treatments for chronic non-healing wounds has been of great interest for many years. To date, there are numerous methods and strategies for treating chronic non-healing wounds (Han and Ceilley, 2017; Okur et al., 2020). Non-etheless, since chronic wound healing is a complex and long-term process involving inflammatory response, angiogenesis, re-epithelialization, and collagen deposition, the therapeutic effects of current treatments are limited and unsatisfactory (Table 1) (Zielins et al., 2014; Boateng and Catanzano, 2015). Therefore, novel curative therapies for chronic non-healing wounds need to be explored.

**TABLE 1:** Current chronic wound treatments.

Current therapies	Advantages	Limitation	Ref
Debridement	Remove necrotic tissue, prevent infection	Lack of nutrition and blood supply, trauma	Jones et al. (2018)
Hyperbaric oxygen therapy	Prevent infection, boost angiogenesis	Lack positive effects on wound healing and amputation rates	Ruemenapf et al. (2022)
Antibiotics therapy	Prevent infection	Drug adverse effects, drug resistance	Bjarnsholt et al. (2008)
Flap transplantation	Re-vascularization	Multi-risk, trauma, low survival rate	Demmer et al. (2021)
Functional active dressing	Prevent infection, promote granulation forming, provide moist and suitable microenvironment	Lack of adequate clinical data, high costs, unsolved underlying pathophysiologic disorder around	Jones et al. (2018)
Vacuum sealing drainage technology	Prevent infection, increase blood supply, decrease wound size and discomforts		
Tissue engineering skin	Good histocompatibility, non-immunogenic, maintain normal morphology and function		

A large body of evidence has demonstrated that mesenchymal stem cells (MSCs) derived from a variety of tissues, possess great therapeutic potentials for chronic non-healing wound treatment by regulating multiple processes such as inflammatory response, angiogenesis, re-epithelization, extra cellular matrix (ECM) remodeling and scarless healing (Kosaric et al., 2019; Mazini et al., 2020; Fageeh, 2021). Nevertheless, there are many limitations and obstacles in the direct application of MSCs, such as tumorigenicity (Jeong et al., 2011), immune rejection (Ankrum et al., 2014), and different cellular features (Conrad et al., 2009; Haga et al., 2015). Therefore, researchers still strive to develop a novel cell-free therapy for chronic non-healing wound treatment. Recently, several studies disclosed that MSCs act on chronic wound healing mainly through their paracrine function rather than their ability to differentiate into skin cells at wound sites (Cao et al., 2017). Small extracellular vesicles (sEVs) or exosomes are enriched in the secretome of MSCs. Thus lately, there is a rush to explore the role of MSC-sEVs in wound treatment (Yaghoubi et al., 2019; An et al., 2021; Vu et al., 2021) so as to optimize the application of sEVs as the substitute of cellular therapy with MSCs. sEVs were first described in the 1970s by Johnstone who separated them from sheep reticulocytes (Johnstone et al., 1987). In the past, sEVs were ignored and thought of as cellular dust. Today, scientists increasingly realize that sEVs carrying intercellular biological information are promising biological tools for treatment of a variety of diseases (Panfoli et al., 2018). Particularly, recent studies have reported that MSC-sEVs accelerate chronic wound healing by regulating and restoring normal cellular behavior at wound sites (Casado-Díaz et al., 2020).

In this review, we describe the characteristics of MSCs and MSC-sEVs and discuss their therapeutic potential and application avenues in chronic wound treatment. Additionally, strategies for large-scale production and engineering of MSC-sEVs were summarized, which contribute to obtaining large quantities and specific functionalized MSC-sEVs for clinical applications on chronic non-healing wound treatment.

## 2 Biological characteristics and clinical applications of MSCs

### 2.1 Biological characteristics of MSCs

**FIGURE 2:**
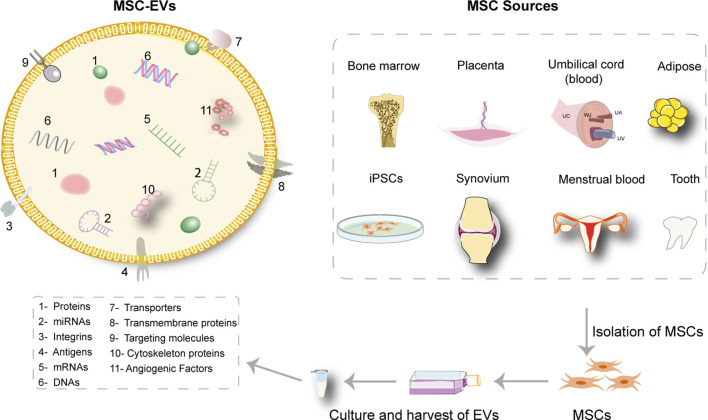
The content of MSC-sEVs and extensive sources of MSCs. The isolated sEVs from MSCs are enriched in a large variety of therapeutic molecules. The sources of MSCs are extensive including bone marrow, umbilical cord, placenta, adipose tissue, synovial membrane, exfoliated deciduous teeth, menstrual blood, and induced pluripotent stem cells (iPSCs).

MSCs are multi-potent adult stem cells possessing multi-lineage differentiation capacity and immunosuppressive properties. The sources of MSC are very extensive (Jo et al., 2021) and they can be harvested from bone marrow, umbilical cord, adipose tissue, synovial membrane, gingiva, and some unconventional sources including exfoliated deciduous teeth, menstrual blood, and fetal dermis of accidentally aborted fetuses (Figure 2) (Wang et al., 2019a; Dalirfardouei et al., 2019; Narbute et al., 2019). Accordingly, MSCs should express CD105, CD73, and CD90, with lack of expression of CD34, CD45, CD14 or CD19, CD79a or CD11b, and HLA-DR. Other markers including CD146, CD271, Stro-1, and SSEA-4 are reported to relate to the stemness and sources of MSCs (Lv et al., 2014). Criteria for isolation, culture, and identification of MSCs have been established by the International Society for Cellular Therapy (ISCT) (Galderisi and Giordano, 2014). The biological roles of MSCs in skin regeneration and repair have been reported widely (Kosaric et al., 2019; Zhang et al., 2020; Malhotra et al., 2021). Collectively, the biological roles of MSCs include controlling excessive immune response in skin wounds for their immunomodulatory capacity (Uchiyama et al., 2017), secreting paracrine factors such as EVs to contribute to the healing process (Qi et al., 2014; Bian et al., 2020), and differentiating into skin repair cells for their multiple differentiation potential (Zhang et al., 2020).

### 2.2 Clinical applications of MSCs in wound treatment

MSCs have been frequently used and examined in clinic. For skin wound treatment, a series of clinical trials were performed to evaluate the efficacy of MSC application in serious skin burns (Falanga et al., 2007), non-healing ulcers (Dash et al., 2009; Li et al., 2013; Chen et al., 2018a; Lu et al., 2019; Moon et al., 2019; Kerstan et al., 2021; Zhang et al., 2022a; Kerstan et al., 2022) and advanced limb ischemia (Yang et al., 2013; Lu et al., 2019). For example, Sheng et al. first reported successful employment of MSCs to realize the regeneration of functional sweat glands in patients with deep burn injury (Sheng et al., 2009). Besides, Lu and colleagues showed that application of autologous BM-MSCs in forty-one patients with DFUs could lead to improved ulcerative healing rate and blood infusion, decreased ulcer relapse and amputation during a 3-year follow-up (Lu et al., 2019). In the light of registered trials on https://www.clinicaltrials.gov, more than three hundred clinical trials based on MSC therapy have been finished in patients with including but not only limited to autoimmune or degenerative diseases (Zhou et al., 2021). Here, we listed the clinical trials in patients with non-healing wounds in Table 2. On the whole, MSCs have been proven to have tolerable safety profile and effectiveness in certain clinical settings. However, a lack of verification on safety and therapeutic benefit from large-scale clinical trials barricades the transfer from bench to bedside, impelling researchers to find superior substitute such as MSC-sEVs.

**TABLE 2:** Clinical trials of MSC therapy in skin non-healing defects.

NCT number	Conduct/publication time	Human cell type	Disease	Phase	Object number	Time frame	Clinical parameters	Ref
03,267,784	2017–2020	Allogenic - ABCB5+ MSC	Diabetic neuropathic ulcer	Ⅰ, Ⅱ	23	12 weeks	Diminish wound surface area	Kerstan et al. (202); Kerstan et al. (2022)
Unreported	2007	Autologous-BM-MSC	Acute and chronic wounds (over 1 year)	—	13	20 weeks	Stimulate the healing process	Falanga et al. (2007)
Unreported	2009	Autologous-BM-MSC	Chronic wounds of lower limb	—	24	12 weeks	Reduce wound size, elongate pain-free walking distance, ameliorate blood perfusion of low limb	Dash et al. (2009)
00,955,669	2009–2010	Autologous-BM-MSC	Diabetic Critical Limb Ischemia and DFU	Ⅰ	41	3 years	Boost leg blood perfusion and ulcerative healing, diminish amputation and ulcer recurrence	Chen et al. (2018a); Lu et al. (2019)
ChiCTR 2200055885	2009–2020	Allogenic UCB-MSC	PAD and incurable DFU	Ⅰ	14	3 years	Quicken ulcer closure process, no relief of angiostenosis	Zhang et al. (2022a)
Unreported	2013	Allogenic UCB-MSC	DFU	—	15	12 weeks	Relieve pain, numbness and coldness, improve ABI and TcO2, reduce blood glucose level and amount of required insulin	Li et al. (2013)
Unreported	2013	Allogenic UCB-MSC	PAD	Ⅰ	8	6 months	Ulceration healed in three of four, angiographic scores added in three of eight	Yang et al. (2013)
02,619,877	2015–2016	Allogenic AD-MSC	DFU	Ⅱ	59	12 weeks	Accelerate wound closing and re-epithelialization	Moon et al. (2019)

BM, bone marrow; UCB, umbilical cord blood; DFU, diabetic foot ulcer; PAD, peripheral arterial disease.

## 3 Biological characteristics of MSC-sEVs

Extracellular vesicles (EVs) are defined as naturally released double-layered membrane particles or vesicles (Théry et al., 2018). Currently, there is still no consensus about the specific markers of EV subtypes. Herein, we endorsed the term small EVs (sEVs) with ranges defined no more than 200 nm based on minimal information for studies of extracellular vesicles 2018 (MISEV 2018) (Théry et al., 2018). Of note, a great deal of literature has endorsed the term “exosomes,” which are defined as sEVs with a size range from 40–160 nm and exosomes have been the interest of their studies (Kalluri and LeBleu, 2020). Therefore, in the present review, the term of “sEVs” is employed to refer to small EVs and exosomes. In this section, will briefly introduce features of sEVs, advantages of MSC as the producers of sEVs and isolation and purification of sEVs. Biogenesis or uptake of sEVs is not covered here, but has been reviewed previously (Bian et al., 2019; Pegtel and Gould, 2019; Lv et al., 2020).

### 3.1 Characteristics and composition of sEVs

sEVs are predominately characterized by microstructures, sizes, and surface makers. Investigators mainly observe the vesicle-like or cup-like microstructure of sEVs with the help of transmission electron microscopy (Gurunathan et al., 2019; Jafari et al., 2020). Meanwhile, the size of sEVs could be examined by nanoparticle tracking analysis (NTA) or tunable resistive pulse sensing (Sokolova et al., 2011). Together with typical structure and size, sEVs have exclusive markers on their surfaces. One type of the surface proteins are ESCRT-associated proteins, such as tumor suppressor genes (TSG101) and ALG-2 interacting protein X (Alix). While others are ESCRT-independent markers (CD9, CD63, CD81), flotillin, chaperones, and ubiquitinated proteins (de Gassart et al., 2003; Monguió-Tortajada et al., 2019). However, specificity of some markers (such as RACGAP1) and typical subtype markers to distinguish the subtypes of EVs are still debated, calling for more studies to unveil these doubts in the future (Xu et al., 2017).

sEVs are mainly composed of lipids, proteins, and nucleic acids including mRNAs, non-coding RNAs, and DNAs (Keerthikumar et al., 2016) and therefore are thought to be carriers of intercellular biological information. Furthermore, the biochemical content of sEVs varies according to their origin, and thus, the biological information they carry also differs. The heterogeneity of sEV composition is possibly reflective of their origins, sizes, functional impacts on acceptor cells. Reportedly, MSC-sEVs are also enriched in various bioactive molecules which have good therapeutic effects on diverse diseases (Simons and Raposo, 2009).

### 3.2 MSCs: Source of sEVs

Furthermore, employment of MSCs and their derivatives has gained momentum in the field of regenerative medicine (Malhotra et al., 2021). A line of basic and clinical trials have demonstrated that MSCs can create a beneficial microenvironment to regulate inflammatory response, facilitating the formation of a properly-vascularized granulation matrix, enhancing the proliferation and migration ability of skin cells and diminishing apoptosis, thus accelerating wound healing (Mazini et al., 2020). MSC-sEVs have also been verified to be therapeutic in preclinical studies and exert immune-modulating and regenerative effects (Bian et al., 2019). Of note, the establishment of immortalized MSCs impacts on neither the quality nor the quantity of MSC-sEVs. Besides, the immortalized treatment is beneficial for its sustainable and cloneable manufacture (Yeo et al., 2013). Compared with other stem cells, MSCs are safer and more favorable in allogenic application, which is evidenced by various clinical trials (Xiao et al., 2013; Squillaro et al., 2016; Bartolucci et al., 2017; Galipeau and Sensébé, 2018; Ban et al., 2021). Accordingly, we speculate that sEVs from MSCs are also safer than that of other stem cells for allogenic transplantation.

### 3.3 Isolation and purification of sEVs

Ultracentrifugation-based isolation has been most widely employed and considered to be the golden technique for sEVs separation on account of accessibility, simplicity, high yield, and harvest of comparatively homogenous size groups of sEVs, yet it is lengthy and labor-intensive and might lead to co-sedimentation of non-vesicular proteins (Pegtel and Gould, 2019; Tian et al., 2020). To fix the disadvantages of routine sEV isolation, multiple techniques based on different rationales have been used, such as ultrafiltration, polymer-based precipitation, immunoaffinity capture, size exclusion chromatography, and combined employment of the techniques (Tian et al., 2020). Ultrafiltration provides a faster substitute to ultracentrifugation. However, the particle yield and purity might be compromised because of absorption to cellulose and membranes and deformation of vesicles owing to pressure (Lobb et al., 2015; Konoshenko et al., 2018). Thus, ultrafiltration might be more suitable with limited liquid volume for clinical grade sEVs. As another suitable technique under clinical research conditions, polymer-based precipitation is also time-efficient, low-cost and leads to a high yield. The non-specific mechanism of it also brings about shortcomings including low quality of sEVs and co-sedimentation of non-vesicular contamination (Sidhom et al., 2020). Notably, immunoaffinity capture can harvest sEVs of higher purity but lower yield and is extensively applied as an adjuvant step with ultracentrifugation to enhance pureness. Drawbacks of this method include selecting one subtype of sEVs, not capturing all sEVs, being expensive, and confining to small sample size (Sidhom et al., 2020). Accumulating data have suggested that size exclusion chromatography is superior in purification of sEVs, which is exceptional in preservation of functionally and morphologically intact sEVs. But researchers should prevent biological target denaturation and take the sample volume into consideration when employing this method (Stulík et al., 2003; Sidhom et al., 2020).

Regrettably, different extraction and purification methods of sEVs may produce different populations of sEVs carrying various functional cargoes including proteins and nucleic acids. Additionally, some methods can also lead to the contamination of sEVs with non-vesicular “contaminants.” Above technical limitations likely bring about contradictory or unrepeatable conclusions in the treatment of sEVs on diseases including non-healing wounds. Therefore, we believe that combined employment of the techniques is probably the best choice to obtain highly purified and intact sEVs which can perform in a replicable manner.

## 4 Therapeutic potential of MSC-sEVs for chronic non-healing wound treatment

**FIGURE 3:**
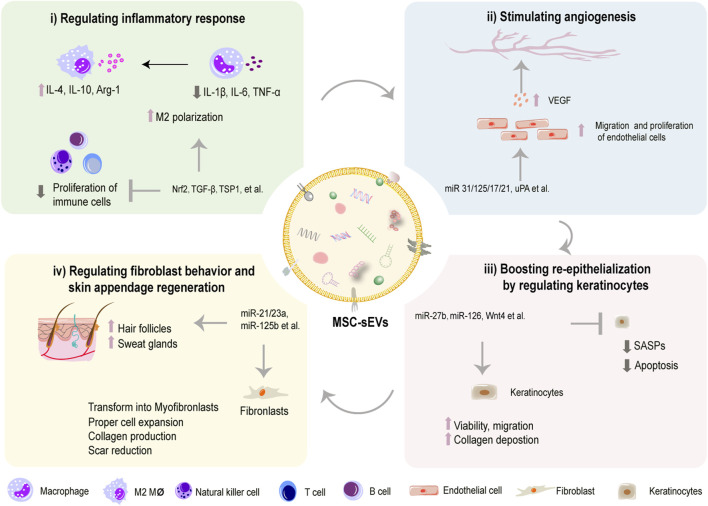
MSC-sEVs promote wound healing process by multiple mechanisms. MSC-sEVs can regulate inflammatory response by inhibiting proliferation of immune cells and shifting polarization of macrophages (i). MSC-sEVs stimulate angiogenesis by promoting VEGF-mediated migration and proliferation of endothelial cells (ii). MSC-sEVs boost re-epithelization by inhibiting SASPs and apoptosis and increasing the viability and migration of keratinocytes (iii). MSC-sEVs inhibit scar formation by modulating transformation of fibroblasts to myofibroblasts (iv).

For treatment of non-healing wounds, the severity of the wounds and overall health status will presumably affect the effects and outcomes of MSC-sEV treatment in clinical settings. Therefore, we might propose that effects of treatments on DFUs of different clinical grades of different groups should be evaluated respectively in clinical trials of MSC-sEV. It has been proved that MSC-sEVs can function on many stages of wounds (An et al., 2021). In this section, as briefly demonstrated in Figure 3, we will discuss the therapeutic potencies and underlying mechanisms of employment of multiple sourced MSC-sEVs to facilitate chronic non-healing wound by regulating and restoring cellular functions at wound sites.

### 4.1 Effects of MSC-sEVs on hemostasis stage

The wound healing cascade starts with hemostasis. The immediate response is vasoconstriction of the injured blood vessels to stop bleeding, followed by platelet aggregation, platelet plug formation and the coagulation cascade activation to form a fibrin clot which halts the blood flow and offers a scaffold for inflammatory cells (Rodrigues et al., 2019). Notably, data about the application of MSC-sEVs in hemostasis phase of the wound healing process is limited. It has been reported that MSC-sEVs possess procoagulant activity *in vitro*. Specifically, Tiffani et al. demonstrated that EVs, either from AD-MSCs or BM-MSCs, were functionally thrombogenic and likely to enhance clotting rates by expressing tissue factor and phosphatidylserine on their surfaces (Chance et al., 2019). So, we have reasons to expect potential effects of MSC-sEVs in hemostasis stage of wound healing process *in vivo* in the future.

### 4.2 Regulating inflammatory cells

Generally, inflammatory phase, which occurs at several minutes to hours after skin injury and lasts for several days, stands at the beginning stage of the healing cascade. A well-regulated inflammatory response is essential to trigger healing process of skin wounds. However, malfunction of immune cells, such as macrophages, T lymphocytes, and B lymphocytes, is regarded as the culprit to generate persistent excessive inflammation, which is the typical characteristic of chronic non-healing wounds (Li et al., 2021). Accumulating evidence has suggested that MSC-sEVs exert anti-inflammatory and immunomodulating effects on those cells thus facilitating wound healing (Ti et al., 2015; Monguió-Tortajada et al., 2017; He et al., 2019; Ha et al., 2020). They are able to convert pro-inflammatory M1 macrophages predominately into anti-inflammatory M2 phenotype (Figure 3i) (Ti et al., 2015). Besides, Khare et al. (2018) found that MSC-sEVs from bone marrow could regulate the activation and differentiation of B lymphocytes and inhibit proliferation of some types of immune cells, which may help pave the way for resolution of prolonged inflammation. Also, they could suppress the sensitization and proliferation of natural killer cells to alleviate excess inflammation (Fan et al., 2019). Meanwhile, over-abundant inflammatory cytokines are also common factors for extensive inflammation. Studies have demonstrated that sEVs harvested from bone marrow MSCs (BM-MSCs) pretreated with nuclear factor erythroid related factor 2 (Nrf-2) reduced wound inflammation in diabetic rats by downregulating pro-inflammation cytokines like tumor necrosis factor-α (TNF-α) and interleukin-1β (IL-1β) as well as upregulating anti-inflammation factors such as IL-4 and IL-10 (Wang et al., 2021a). Mechanically, functional cargos, such as non-coding RNAs (Fatima et al., 2017), are listed in Table 3 (Li et al., 2020). Therefore, MSC-sEVs may provide a clinically applicable method in alleviating overwhelmed inflammation in chronic non-healing wound.

**TABLE 3:** MSC-sEVs play an active role in regulating inflammatory cells.

MSC source	Isolation	Target cells/conditions	Functional cargo	Molecules/Pathways affected	Key functions/Downstream genes	Reference
BM	precipitation	cutaneous wound in mice; human monocytes *in vitro*	miR-223	Pknox1**↓**	enhanced M2 polarization, TNF-α↓, IL-10↑, Arg-1↑	He et al. (2019)
Huc	Size-Exclusion Chromatography	T cell, PBMCs	—	—	reduced T cell proliferation	Monguió-Tortajada et al. (2017)
BM	Ultracentrifugation	PBMC, T and B cells	—	—	Decreased proliferation of PBMC, T and B cells and IgM levels	Khare et al. (2018)
Fetal liver	ultracentrifugation	NK cells	LAP, TGFβ and TSP1	p-SMAD2/3↓	Restrained proliferation, function and activity of NK cells	Fan et al. (2019)
LPS-pre-huc	ultracentrifugation	Diabetic cutaneous wound in rats; THP-1 *in vitro*	let-7b	TLR4↓, p-P65↓, NF-κB↓, p-STAT3↑, p-AKT↑	Skewed macrophage polarization to M2 and clearance of chronic inflammation	Ti et al. (2015)
Menstrual blood	Ultracentrifugation	cutaneous wound in diabetic mice	—	ARG/iNOS↑	Elevated M2/M1 ratio and resolved inflammation	Dalirfardouei et al. (2019)
Murine BM	ultracentrifugation	DFU model in mice; HDF	LncRNA H19	miR-152-3p↓ → PTEN↑ → p85PI3K↓→p-AKT↓→IL-1β↓, TNF-α↓, and IL-10↑	suppressed apoptosis and inflammation *in vitro* and *in vivo*	Li et al. (2020)
BM	Ultracentrifugation	EPC; cutaneous wound in diabetic rats	Nrf2	IL-1β↓, TNF-α↓and IL-10↑, IL-4↑	Reduced inflammation	Wang et al. (2021a)

Huc, human umbilical cord; BM, bone marrow; Pknox1, PBX/knotted 1 homeobox one; PBMCs, Peripheral Blood Mononuclear Cells; LAP, latency associated peptide; TGFβ, transforming growth factor β; TSP1, thrombospondin 1; ARG, arginase; THP-1, human myeloid leukemia mononuclear cells; TLR4, Toll-like receptors 4; DFU, diabetic foot ulcer; AT, adipose tissue; Nrf2, nuclear factor erythroid 2–related factor 2; EPC, endothelial progenitor cell; SMP30, senescence marker protein 30; NOX1/4, NAPDH, oxidase 1/4; TNF-α, Tumor Necrosis Factor α; hAAM, human acellular amniotic membrane.

### 4.3 Stimulating angiogenesis by regulating vascular endothelial cells

Angiogenesis is crucial in promoting wound healing and skin repair in terms of transportation of oxygen, nutrients, and immune cells to wounded sites, facilitating cell proliferation and ECM deposition (Wang et al., 2019b). Although healing process is influenced by many factors, poor vascularization may be the most principle cause for wound chronicity (Cheng and Fu, 2018). Furthermore, the depth causes for insufficient angiogenesis could be the undue inflammatory, excessive oxidative stress, DNA damage, and cell cycle arrest, leading to cell senescence and impairing pro-angiogenic ability of endothelial cells (Pulido et al., 2021). A line of evidence has suggested that MSC-sEVs can enhance proliferation and migration of vascular endothelial cells, thus promoting angiogenesis and blood vessel maturation in diabetic wounds (Figure 3ii) (Shabbir et al., 2015; Kang et al., 2016; McBride et al., 2017; Li et al., 2018; Shi et al., 2020; Guillamat-Prats, 2021; Wei et al., 2021). As concluded in Table 4, these desirable effects were associated with elevated expression of genes involved in proliferation (PCNA, cyclin D3), migration (Konoshenko et al., 2018), and angiogenesis (VEGF, Ang1, and Flk1) (Zhang et al., 2015a; Liang et al., 2016; Hu et al., 2018). At a molecular level, a group of non-coding RNAs are detected to enriched in MSC-sEVs, such as HOTAIR (HOX transcript antisense RNA), miR-221-3p, −21, −125a, −17, and −126, which have been proved to mediate favorable bioeffects by regulating above target genes (Tao et al., 2017; An et al., 2019; Yu et al., 2020; Born et al., 2021; Wei et al., 2021; Pi et al., 2022). For instance, we previously found that miR-17-5p in umbilical cord MSC-sEVs (hucMSC-sEVs) accelerated angiogenesis in diabetic mice *via* targeting PTEN/AKT pathway, thus exerting positive effects on wound healing (Wei et al., 2021). Besides, application of MSC-EVs has also been demonstrated to be efficacious in mitigating excessive oxidative stress and senescence-associated secretory phenotype of endothelial cells (Wang et al., 2020). To be specific, Zhang and coworkers reported that adipose sourced MSC-sEVs could alleviate oxidative stress, diminish reactive oxygen species (ROS) production and ameliorate mitochondrial function in endothelial cells under high glucose by regulating Sirtuin 3 (SIRT3)/superoxide dismutase 2 (SOD2) activity, thereby achieving better vascularization and diabetic wound healing (Sidhom et al., 2020). Likewise, Xiao et al. found that the treatment with MSC-EVs effectively improved the senescence of HG-treated endothelial cells in diabetic wounds *via* regulating miR-146a/Src pathway, leading to a decrease in aging-related proteins p21/p16/p53 and an enhanced angiogenesis in diabetic wounds (Xiao et al., 2021). Not only RNAs but also active proteins in MSC-sEVs can stimulate angiogenesis in wound restoration (Sung et al., 2019; Tutuianu et al., 2021). For instance, Angiopoietin-2 (Ang2) and deleted in malignant brain tumors 1 (DMBT1) have been reported to be abundant in MSC-sEVs to exert pro-angiogenic functions during wound healing (Chen et al., 2018b; Liu et al., 2021). Loaded with angiogenic components, MSC-sEVs are expected to be a prominent option to induce angiogenesis to treat poorly vascularized wounds in future clinical practice.

**TABLE 4:** MSC-sEVs stimulate angiogenesis by regulating vascular endothelial cells in wound healing.

MSC source	Isolation	Target cells/conditions	Functional cargo	Molecules/Pathways affected	Key functions/Downstream genes	Ref
BM	Ultracentrifugation	HUVECs (normal and chronic wounds)	STAT3	p-ERK1/2↑, p-Akt↑, p-STAT3↑, HGF↑, IGF1↑, NGF↑, SDF1↑	enhancement of proliferation and migration HUVECs	Shabbir et al. (2015)
AT	Ultracentrifugation	HUVECs	MiR-31	FIH1↓, CD31↑	Enhanced angiogenic ability of HUVECs	Kang et al. (2016)
Nrf2-OE-AT	Co-Precipitation	EPC *in vitro*, diabetic foot ulcer in rats	Nrf2	SMP30↑, VEGF↑, P-VEGFR2/VEGFR2↑	Promoted cell viability, migration and angiogenesis both *in vitro* and *in vivo*	Li et al. (2018)
Urinary	Ultrafiltration	cutaneous wound in diabetic mice; HMECs	DMBT1	VEGFA↑, p-AKT↑, CD31↑	Elevated angiogenic responses *in vitro* and angiogenesis *in vivo*	Chen et al. (2018b)
BM	Polymer precipitation; Ultracentrifugation	HUVECs	Wnt3a	—	Enhanced proliferation, migration and angiogenesis *in vitro*	McBride et al. (2017)
AT	Ultracentrifugation	HUVECs immunodeficient mice	MiR-125a	DLL4↓, Ang1↑, Flk1↑	Activated angiogenesis *in vitro* and *in vivo*	Liang et al. (2016)
Modified-AT	Ultrafiltration Ultracentrifugation	Diabetic wounds in mice, EPC	mmu_circ_0000250	miR-128-3p↓, SIRT1↑	Activated autophagy and proangiogenic abilities and suppressed apoptosis *in vitro*; increased neovascularization *in vivo*	Shi et al. (2020)
Modified synovium	Ultracentrifugation	cutaneous wound in diabetic rats; HMECs	MiR-126	p-AKT↑, p-ERK1/2↑	Stimulated angiogenesis *in vivo*; activated proliferation, migration and tube formation of HMEC-1	Tao et al. (2017)
huc	Ultracentrifugation	deep second-degree burn injury in rats; HUVECs	Ang-2	CD31↑	Enhanced migration and tube formation of HUVECs and angiogenesis *in vivo*	Liu et al. (2021)
huc	Ultracentrifugation	cutaneous wound in diabetic mice; HUVECs	Wnt4	PCNA↑, cyclin D3↑, N-cadherin↑, β-catenin↑, E-cadherin↓	Elevated proliferation, migration and angiogenic abilities of HUVECs; activated neovascularization *in vivo*	Zhang et al. (2015a)
Modified AT	affinity chromatography	HUVECs	MiR-21	PTEN↓, p-AKT↑, p-ERK1/2↑, HIF-1α↑, SDF↑,VEGFA↑	stimulated vascularization	An et al. (2019)
Thrombin pretreated hucb	Ultracentrifugation	cutaneous wound in rats; HUVECs	angiogenin, angiopoietin-1, HGF, VEGF	p-ERK1/2↑, p-AKT↑	Enhanced proangiogenic activity *in vitro* and accelerated neovascularization and cutaneous wound healing *in vivo*	Sung et al. (2019)
Huc	Ultracentrifugation	cutaneous wound in diabetic mice; HUVECs	miR-17-5p	PTEN↓, p-AKT↑, HIF-1α↑, VEGF↑	Boosted proliferation and migration, tube formation of HUVECs and neovascularization *in vivo*	Wei et al. (2021)
ATV-pretreated BM	Ultracentrifugation	Skin wounds in diabetic rats, HUVECs	MiR-221	PTEN↓, p-AKT↑, p-eNOs↑, VEGF↑	Promoted proliferation and migration activity of HUVECs and neovascularization *in vivo*	Yu et al. (2020)
HOTAIR-OE-BM	Ultracentrifugation	Skin wound in rats and diabetic mice; HUVECs, HMECs	Lnc HOTAIR	VEGF↑	Improved angiogenesis and accelerated wound healing, boosted pro-angiogenic activities of endothelial cells *in vitro*	Born et al. (2021)
BM	Ultracentrifugation	3D human Skin Organotypic model; ECs	Ang2, ET-1, EG-VEGF/PK1, Persephin, uPA		Promoted angiogenesis in model and enhanced angiogenic ability *in vitro*	Tutuianu et al. (2021)
AT	Ultracentrifugation	HUVECs, cutaneous wounds in aged and diabetic mice	MiR-146a	p-Src↓, p-VE-cadherin↓, p-caveolin-1↓, p21↓, p16↓, p53↓	Decreased SASP, rescued angiogenesis *in vitro*; promoted neovascularization in wound healing	Xiao et al. (2021)
BM	Ultracentrifugation	EPCs; ischemic hindlimb in aged mice	MiR-126a	Spred-1↓, p16Ink4a↓, CD31↑	Rejuvenation of aged EPCs, attenuated SA-β-Gal expression	Wang et al. (2020)

BM, bone marrow; HUVEC, human umbilical vein endothelial cell;,STAT3, signal transduction and activators of transcription 3; Erk1/2, extracellular regulated kinase 1/2; AKT, protein kinase B; HGF, hepatocyte growth factor; IGF-1, insulin like growth factor 1; NGF, nerve growth factor; SDF1, stromal cell-derived factor1; HiPSC, human induced pluripotent stem cell; FIH1, factor-inhibiting HIF-1; Nrf2, nuclear factor erythroid 2–related factor 2; EPC, endothelial progenitor cell; SMP30, senescence marker protein 30; HMEC, human microvascular endothelial cell; DMBT1, deleted in malignant brain tumors 1; VEGFA, vascular endothelial growth factor A; DLL4, delta-like 4; HGF, hepatic growth factor; VEGF, vascular endothelial growth factor; HOTAIR, HOX, transcript antisense RNA; EC, endothelial cells; Ang-2, angiopoietin-2; ET-1, endothelin; EG-VEGF/PK1, endocrine gland derived vascular endothelial growth factor; uPA, urokinase-type plasminogen activator; hAAM, human acellular amniotic membrane.

### 4.4 Boosting re-epithelization by regulating keratinocytes

Wound healing can be severely retarded by dysfunctional re-epithelization caused by impaired proliferative and migratory capacities of keratinocytes (Berlanga-Acosta et al., 2020). To restore efficient re-epithelialization is indispensable for successful wound healing. As summarized in Table 5, some studies have observed the potency of MSC-sEVs on boosting re-epithelialization in chronic wounds (Figure 3iii) (Zhang et al., 2015b; Ferreira et al., 2017; Sung et al., 2019; Cheng et al., 2020). Specifically, Tutuianu et al. validated regenerative abilities of BMMSC-sEVs. Exposure to these sEVs enhanced the proliferation and migratory abilities of keratinocytes *in vitro* (Tutuianu et al., 2021). Consistently, Wang et al. applied adipose MSC-sEVs encapsuled in an antibacterial polypeptide-based F127/OHA-EPL hydrogel to treat diabetic wounds and observed remarkable acceleration of re-epithelialization and angiogenesis *in vivo* (Wang et al., 2019c). Besides, Zhao et al. found that hucMSC-sEV administration dramatically increased cell proliferation and inhibited apoptosis *via* restraining apoptosis-inducing factor nucleus translocation (Zhao et al., 2020). While some studies postulate that the miRNA related cargoes containing in MSC-sEVs may be responsible for the bioeffects. For example, Gondaliya et al. observed faster re-epithelialization and enhanced wound repair in diabetic mice treated with MSC-sEVs loaded with miR-155 inhibitor *via* accelerating keratinocyte migration and enhancing fibroblast growth factor-7 (FGF-7) level (Gondaliya et al., 2022). Additionally, miR-205 is reported to be involved in cell migration and proliferation. Lack of miR-205 leads to epidermal defects because of impaired cell proliferation (Wang et al., 2013). This microRNA regulates AKT activation and, therefore, promotes migration of keratinocytes and enhances wound healing (Yu et al., 2010). Moreover, the presence of miR-205 was found in sEV samples from adipose tissue MSCs through the Next-Generation Sequencing experiments. However, it was reported that an miR-205-independent activation of AKT was responsible for the migration and proliferation of keratinocytes in skin wound healing after exposure to adipose tissue MSC-derived EVs (Ferreira et al., 2017). Taken together, there is no doubt that beneficial effects of MSC-sEVs are potential and remarkable. But more studies are required to determine the essential functional constituents and to elucidate the complexity of MSC-sEVs from different tissues for further development of application.

**TABLE 5:** MSC-sEVs exert therapeutic effects in boosting re-epithelialization during wound healing.

MSC source	Isolation	Target cells/conditions	Functional cargo	Molecules/Pathways activated	Key functions/Downstream genes	Ref
AT	Ultracentrifugation	Skin wound in rats; HDFs, keratinocytes		p-AKT↑, p-histone H3↑	Stimulated proliferation and migration of skin cells *in vitro*	Ferreira et al. (2017)
Huc	Ultracentrifugation	Deep second-degree burn in rats; HaCaTs, RDFs	Wnt4	β-catenin↑, p-GSK3β↑, p-AKT↑→CK19↑, PCNA↑, Col Ⅰ↑	Enhanced proliferation and migration of skin cells *in vitro*; promoted re-epithelialization *in vivo*	Zhang et al. (2015b)
Huc	Ultracentrifugation	HaCATs, HDFs *in vitro*; skin wounds in mice	MiR-27b	ITCH↓→JUNB↑→ IRE1α↑	improved proliferation and migration of skin cells *in vitro*; improved epidermal re-epithelialization and collagen proliferation	Cheng et al. (2020)
Huc-WJ	Ultracentrifugation	HaCaT, skin wounds in mice		N-AIF↓, M-AIF↑, N-PARP-1↓, PAR↓	Enhanced re-epithelialization and angiogenesis *in vivo*; inhibited apoptosis and increased proliferation and migration of HaCaT	Zhao et al. (2020)
BM	Ultracentrifugation	3D human Skin Organotypic model; HDFs, HaCaT	—	—	Faster re-epithelialization; enhanced proliferation and migratory capacity *in vitro*	Tutuianu et al. (2021)
BM	Differential centrifugation	Keratinocytes *in vitro*; diabetic wound in mice *in vivo*	MiR-155 Inhibitor	FGF-7↑, VEGF↑, MMP-2↓,MMP-9↓, TGF-β1↓, IL-1β↓, IL-6↓, and TNF-α↓	Accelerated re-epithelialization, angiogenesis and collagen deposition	Gondaliya et al. (2022)

AT, adipose tissue; HDFs, human dermal fibroblasts; AKT, protein kinase B; HaCaT, human immortal keratinocyte line; HSF, human skin fibroblast; RDFs, Rat dermal fibroblasts; GSK-3β, glycogen synthase kinase-3β; PCNA, proliferating cell nuclear antigen; PTEN, phosphatase and tensin homolog deleted on chromosome ten; ERK, extracellular regulated kinase; Bax, BCL2-associated X protein; Bcl-2, B-cell lymphoma-2; huc, human umbilical cord; ITCH, Itchy E3 ubiquitin protein ligase; JUNB, Recombinant Jun B Proto Oncogene; IRE1α, inositol-requiring enzyme 1α; HFFs, human foreskin fibroblasts; MMP, matrix metalloprotein; PDGFA, platelet derived growth factor A; AIF, apoptosis-inducing factor; PARP-1, poly ADP, ribose polymerase 1; PAR, poly ADP, ribose; HIF-1α, hypoxia inducible factor-1α; KGF, keratinocyte growth factor; Hes 1, hairy and enhancer of split-1; CXCR4, C-X-C chemokine receptor type 4.

### 4.5 Promoting regenerative healing through optimizing behaviors of fibroblasts

During normal wound healing, fibroblasts proliferate, migrate, and differentiate into myofibroblasts to participate in synthesizing the ECM, to secrete cytokines and growth factors (Dong et al., 2017), and to enhance wound contraction, thereby promoting wound closure (Darby and Hewitson, 2007). However, these capacities of fibroblasts are impaired in chronic wound microenvironments (Wertheimer et al., 2001), which brings about insufficient ECM production and collagen deposition principally in proliferation phase (within 1 month or longer). The mechanism for this impairment includes cellular senescence induced by excessive oxidative stress and advanced glycation end products in diabetic wounds (Bian et al., 2020). Encouragingly, MSC-sEVs are reported to rejuvenate senescent fibroblasts. For example, sEVs from human placental MSCs significantly improved the biological functions of senescent fibroblasts such as promoting their proliferation and migration, enhancing ECM synthesis, and decreasing the overexpression of matrix metalloproteinases (MMPs) (Bian et al., 2020; Zhao et al., 2021). Further study revealed the mechanism for these effects involving inhibiting the expression of receptor for AGEs (RAGE) and stimulating the activation of Smad signaling pathway in these cells (Bian et al., 2020; Zhao et al., 2021). Interestingly, Zhu and colleagues reported that hucMSC-sEVs could not only promote proliferation and migration of fibroblasts, but also remarkedly boost cutaneous nerve regeneration by stimulating fibroblasts to produce nerve growth factors (NGFs), which can stimulate nerve regeneration, re-establish local sensory innervation homeostasis, and enhance angiogenesis, thereby achieving an ideally regenerative healing both morphologically and functionally (Zhu et al., 2022). Additionally, ADSC-Exos can transport miRNAs, lncRNA, and functional proteins (Choi et al., 2018; Cooper et al., 2018; Parvanian et al., 2020; Qian et al., 2021) to promote the migration, proliferation, ECM secretion of fibroblasts (Hu et al., 2016; Wang et al., 2021b).

Pathological scars including keloids and hypertrophic scars arise from excessive wound healing after chronic inflammatory stimulation. The pathological mechanism is the aberrant activation of fibroblasts and myofibroblasts, which synthesize more ECMs in scar tissue, mostly *via* the abnormal activation of transforming growth factor-β (TGF-β)/Smads pathway and Yes-associated protein predominantly during remodeling phase (at least over 14 days to 1 month) (Finnson et al., 2013; Zielins et al., 2014; Song et al., 2018; Clark, 2021). Recently, multiple sourced MSC-sEVs have been proven effective in achieving scarless and high-quality wound regeneration (Figure 3iv) (Wang et al., 2017; Dalirfardouei et al., 2019; Jiang et al., 2020a). Zhang et al. found hucMSC-sEVs treatment worked smartly not only as an activator of the Wnt/β-catenin signaling pathway to heal impaired skin but also an inhibitor of the signal *via* exosomal 14-3-3ζ mediated YAP regulation to avoid scar formation (Zhang et al., 2016). Similarly, in another study, Zhang et al. reported that miR-21-5p and miR-125b-5p in MSC-sEVs from umbilical cord blood played critical roles in suppressing myofibroblast differentiation from fibroblasts, thereby favoring scarless wound healing (Zhang et al., 2021). In line with the same idea, Fang et al. examined miRNA profiles in hucMSC-sEVs by high-throughput sequencing and verified that a couple of miRNAs (miR-21, −125b, −23a and −145) decreased myofibroblast formation *via* suppression of transforming growth factor β (TGF-β) Smad2 pathway (Fang et al., 2016). Encouragingly, similar therapeutic effects were also obtained by another team when they applied MSC-sEVs loaded with tumor necrosis factor-α stimulated gene-6 (TSG-6) or miR-138-5p (Jiang et al., 2020b; Zhao et al., 2022). Additionally, the wounds treated with ADSC-Exos also demonstrated faster wound healing and less collagen deposition (Li et al., 2022). So we conclude that MSC-sEVs exert multiple-mechanism therapeutic effects on wound regeneration (as demonstrated in Table 6).

**TABLE 6:** MSC-sEVs exert curative effects by optimizing fibroblast behavior.

MSC source	Isolation	Target cells/conditions	Functional cargo	Molecules/Pathways affected	Key functions/Downstream genes	Ref
BM	Ultracentrifugation	cutaneous wound in rats; HaCaT, HDFs	—	TGF-β1↓→Smad2/3/4↓; TGF-β3↑, Smad7↑	Improved anti-fibrotic and scar-less wound healing	Jiang et al. (2020a)
Menstrual blood	Ultracentrifugation	cutaneous wound in diabetic mice	—	Col1/Col3↓	Reduced cellularity in granulation tissue and diminished scar formation	Dalirfardouei et al. (2019)
Modified synovium	Ultracentrifugation	cutaneous wound in diabetic rats; HDFs	MiR-126	p-AKT↑, p-ERK1/2↑	Accelerated re-epithelialization and collagen maturity *in vivo*; boosted migration and proliferation of HDFs	Tao et al. (2017)
AT	—	HDFs	miRs (-4484, -619-5p, -6879-5p)	Col Ⅰ↑, Elastin↑, KGF↑, CD34↑, VEGF↑	Boosted proliferation and migration of HDFs	Choi et al. (2018)
BM	Polymer precipitation and ultracentrifugation	HDFs	Wnt3a	—	promoted proliferation and migration abilities	McBride et al. (2017)
Fetal dermal	Polymer precipitation	skin wounds in mice; HDFs	Jagged 1	Notch 1↑ → Hes 1↑→ PCNA↑, CK19↑	Promoted proliferation, migration and secretion abilities *in vitro* and *in vivo*	Wang et al. (2019a)
BM	Ultracentrifugation	3D human Skin Organotypic model; HDFs, HaCaT	—	—	Faster re-epithelialization; enhanced proliferation and migratory capacity *in vitro*	Tutuianu et al. (2021)
Murine BM	ultracentrifugation	DFU model in mice; HDF	LncRNA H19	miR-152-3p↓ → PTEN↑ → p85PI3K↓→p-AKT↓	Improved proliferation and migration and suppressed apoptosis *in vitro* and *in vivo*	Li et al. (2020)
Thrombin pretreated hucb	Ultracentrifugation	cutaneous wound in rats; HDFs	angiogenin, angiopoietin-1, HGF, VEGF	p-ERK1/2↑, p-AKT↑	elevated proliferation and migration activity of HDFs; faster wound healing *in vivo*	Sung et al. (2019)
Huc	Ultracentrifugation	HDFs; skin wounds in mice	miR-21, -23a, -125b, -145	TGF-β2↓, Smad2↓	Suppression of myofibroblasts transformation or scar formation	Fang et al. (2016)
Huc	Ultracentrifugation	HaCATs, MDF; second-degree burn in rats	14-3-3ζ	YAP + p-LAS → p-YAP↑, cytoplasmic retention of YAP↑, a-SMA↓, col I↓ and col III↓	Reduced skin cell proliferation and nuclear translocation of β-catenin under high cell density *in vitro*; restricted excessive cell expansion and collagen deposition during remodeling period *in vivo*	Zhang et al. (2016)
AT	Polymer precipitation	skin wounds in mice; HDF		ERK/MAPK↑→MMP3/TIMP1↑, TGF-β3/TGF-β1↑, Col Ⅲ/Col Ⅰ↑	Mitigated myofibroblast differentiation; optimized ECM remodeling and lessened scar formation *in vivo*	Wang et al. (2017)
TSG-6 modified BM	Polymer precipitation	skin wounds in mice	TSG-6	TGF-β1↓, p-SMAD2^Ser467^/3^S423/S425^↓→ col I↓, col III↓, α-SMA↓; MCP-1↓, TNF-α↓, IL-1β↓, IL-6	prevented inflammation and collagen deposition, restricted scar formation *in vivo*	Jiang et al. (2020b)
Huc	ultracentrifugation	Skin wound in rats; HDF	miR-21-5p, -125b-5p	TGFBR1↓, TGFBR2↓, α-SMA↓, collagen I↓	Suppressed myofibroblast differentiation and scar formation, improved regenerative healing	Zhang et al. (2021)

BM, bone marrow; HaCaT, human immortal keratinocyte line; HDF, human dermal fibroblast; TGF-β, transgenic growth factor β; Huc, human umbilical cord; MDFs, mouse dermal fibroblasts; YAP, Yes-associated protein; LAS, large tumor suppressor; AT, adipose tissue; Erk, extracellular regulated kinase; MMP, matrix metalloproteinase; TIMP, tissue inhibitor of metalloproteinase; MCP, monocyte chemoattractant protein-1; TNF-α, tumor necrosis factor-α; TGFBR, TGF-β, receptor type II.

### 4.6 Promoting regeneration of skin appendages

Most human skin wounds heal with scar and without skin appendages including hair follicles and sweat glands. This outcome seriously affects the appearance of patients and leads to the damage of skin physiological function. Recently, MSC-sEVs were reported to entice regeneration of hair follicles (Table 7). ADSC-EVs containing a variety of cytokines stimulated hair follicle growth by activating the Wnt signaling pathway which is essential to hair follicle induction (Zhang et al., 2022b). Wnt3a is enriched in sEVs from hBMMSCs and BMMSC-sEVs can activate the Wnt/β-catenin signaling in recipient dermal papilla (DP) cells (Rajendran et al., 2022). Our research also implied the hair follicle regeneration potential of sEVs from DP cells (Zhang et al., 2022c). Another group of researchers have successfully designed miR-218-5p abundant sEVs derived from dermal papilla cells and observed a notably promoted hair follicle development by up-regulating β-catenin in mice (Hu et al., 2020). Therefore, MSC-sEVs promote the regeneration of hair follicles mainly by the activation of Wnt/β-catenin pathway (Hu et al., 2020). Additionally, Chen et al. also observed that TGF-β1-enriched MSC-sEVs could realize a faster reconstruction of sweat gland function in wounded skin (Chen et al., 2022a).

**TABLE 7:** MSC-sEVs enhance regeneration of skin appendages.

MSC source	Isolation	Target cells/conditions	Functional cargo	Molecules/Pathways affected	Key functions/Downstream genes	Ref
Human BM	ultracentrifugation	dermal papilla (DP) cells, outer root sheath (ORS) cells from patients with androgenic alopecia	Wnt3a	Wnt/β-catenin↑	Promoted proliferation and activation of DP cells, upregulated proliferation and migration of ORS cells, enhanced hair growth	Rajendran et al. (2022)
Dermal papilla	Ultrafiltration	Shaved skin in mice	miR-218-5p	β-catenin↑, CD133↑, Ki67↑	Promoted hair growth	Hu et al. (2020)
Huc	Ultracentrifugation	Wounded skin and paw in mice; keratinocytes	TGF-β1	E-cadherin↓, a-SMA↑, Slug↑, Cxcr4↑, Sox9↑, Lgr5↑,Oct4↑	Quickened wound healing and sweat gland restoration; enhanced migratory and stem cell properties of keratinocytes	Chen et al. (2022a)

MDFs, mouse dermal fibroblasts; Col Ⅰ/Ⅲ, collagen Ⅰ/Ⅲ; MMP, matrix metalloprotein; TIMP, tissue inhibitor of metalloproteinase; SA-β-Gal, senescence-associated-β-gal; RAGE, receptor for advanced glycation end products; SASP, senescence-associated secretory phenotype; AT, adipose tissue; HUVEC, human umbilical vein endothelial cell; Src, non-receptor tyrosine kinase C; EPC, endothelial progenitor cell; Spred-1, sprouty-related protein 1.

## 5 Application avenues of MSC-sEVs on wounds

**FIGURE 4:**
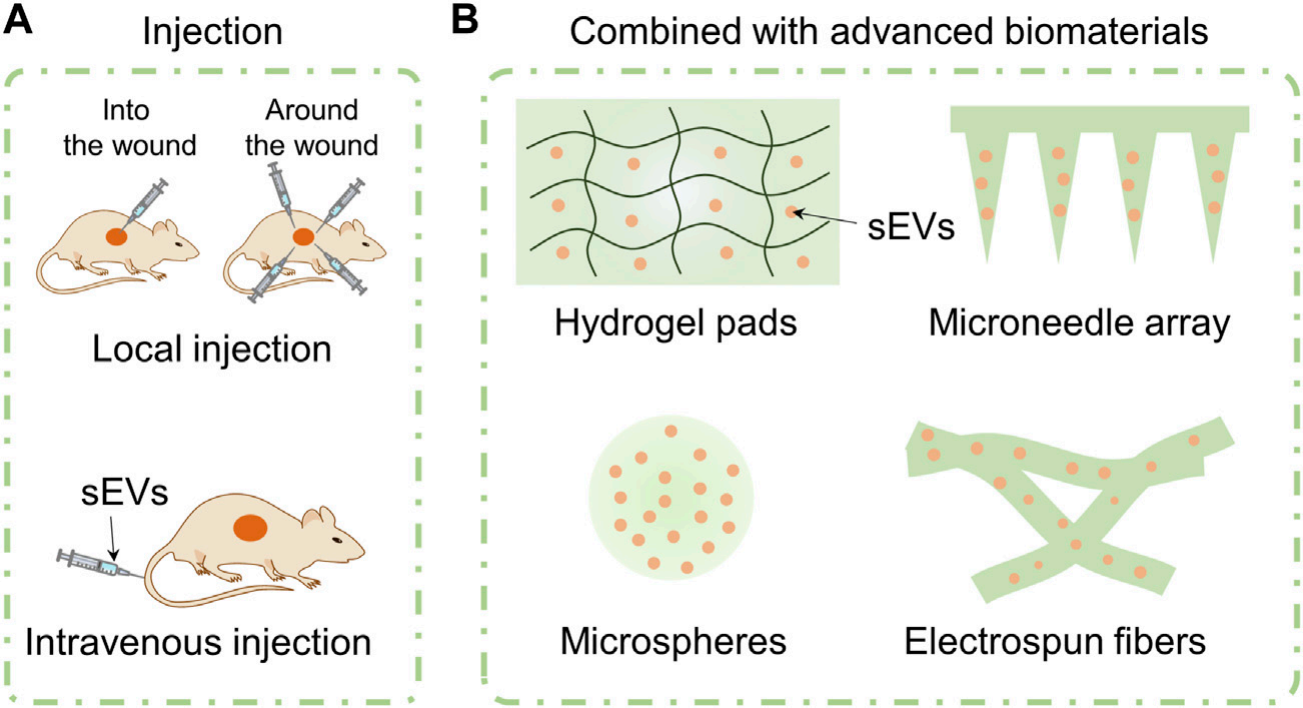
Application avenues of sEVs including injection of free sEVs and combination with advanced biomaterials. Free sEVs are applied by means of local injection or intravenous injection **(A)**. MSC-sEVs are loaded in hydrogel pads, microneedle array, microspheres, and electrospun fibers **(B)**.

Because of their native advantages including anti-inflammatory, angiogenesis, repair promoting, and scar inhibiting mentioned in section 3, MSC-sEVs have been explored to boost wound healing in various forms. Indeed, use of different avenues may affect treatment outcomes of sEVs. In this section, we will focus on the application avenues of sEVs on wounds including injection of free sEVs directly or the combination with advanced biomaterials (Figure 4).

### 5.1 Injection of free sEVs

#### 5.1.1 Local injection

Local injection refers to injecting free sEVs into or around the wounds subcutaneously. Generally, “subcutaneous injection” (Li et al., 2016), “peri-wound injection” (Ti et al., 2015), “intradermal injection at wound edge” (Dalirfardouei et al., 2019), “intra-dermally injection around wound with 4 sites” (Wei et al., 2021) and other terms are used in literatures to refer to the local injection of free sEVs. The injection site is usually in the layer of dermis. After injected locally, sEVs can directly regulate the cell behavior around the wound and improve the wound microenvironment to facilitate healing process. For example, Qiu et al. (2020) reported that injecting sEVs from adult MSCs (AS-MSC-sEVs, 100 μg in 100 μL PBS) pretreated with neonatal serum locally around the wounds of wide type mice with 4 sites every 3 days significantly promoted the cutaneous wound healing. Similarly, we injected hucMSC-sEVs around diabetic wounds of mice at 4 sites (12.5 µL per site) every other day. We observed that injecting locally around the wounds of mice can facilitate healing process of chronic diabetic wounds of db/db mouse through miR-17-5p-mediated enhancement of angiogenesis (Wei et al., 2021). Xia et al. injected sEVs that isolated from young mouse wound-edge fibroblasts (EV-young, 1 μg/μL) around the wounds once a day in an elderly rat and observed wound healing process. They found that sEV-young can induce fibroblasts of elderly rat to transit to myofibroblasts and increase the abundance of myoblasts around wounds (Xia et al., 2022).

Although a large number of literatures reported that local injection of sEVs can facilitate chronic wound healing process. Inconsistent and sketchy description of local injection in these literatures hinders us to analyze that what dose, what administration interval and what injection method of free sEVs can promote chronic wound healing. Additionally, local injection may disturb the wound and cause waste of sEVs.

#### 5.1.2 Intravenous injection

Intravenous administrations including tail vein injection and epicanthus injection are common modes in animal experiments. Using intravenous injection, the bioactive components can enter blood circulation directly, realizing faster drug absorption rate than other methods of administration. Additionally, the therapeutic responses as well as associated toxicity are more predictable than other injection administration (Wong et al., 2008). Dou and the colleagues injected delusional apoptotic bodies (dABs) (100 μg) *via* the tail vein every 2 days after wounding and observed cutaneous wound size every 2 days. The dABs were developed by combing the membrane of ABs and mesoporous silica nanoparticles (MSNs) preloaded with microRNA-21 or curcumin (Dou et al., 2020). Hu et al. (2016) compared the influence of local and intravenous injection of AMSC-sEVs on wound repair process for the first time. Using bioluminescence imaging, the migration and distribution of DIR-labeled AMSC-sEVs (200 μg/μL in PBS) were studied after they were injected into mice suffering a back wound. They found that the fluorescence of AMSC-sEVs gathered at wound site at day 7 and was still detected at day 21 after being injected intravenously. Surprisingly, AMSC-sEVs with intravenous injection showed faster wound closure than local injection group. This phenomenon may be attributed to following reasons. Firstly, local injection of sEVs around the wound will disturb the wound and inevitably destroy the wound healing process. Secondly, local injection may result in the loss of sEVs and reduce their availability. Thirdly, the homing effect of sEVs meditaed by the receptors or adhesion molecules on their membrane surface can regulate the recruiting of sEVs to wound sites.

In our opinions, it is difficult to compare the amount of sEVs and therapeutic effect between local injection and intravenous administration accurately because the content, concentration, and dosing interval of sEVs as well as weight and species of experimental animals used in reported literatures are different from one another. But we can still speculate that using intravenous injection may require fewer exosomes and achieve higher wound closure rate, theoretically. However, there are still application bottlenecks for both injection methods. The rapid clearance of sEVs applied by local injection leads to repeated administration which increases the patient’s suffering. Despite relative long half-time of sEVs applied by intravenous injection, the higher dose (usually higher than 100 μg/μL) of and lower targeting efficacy of sEVs may cause unnecessary waste and potential organo-toxicity. Up to now, several kinds of biomaterials have been used to achieve on-demand release of sEVs, named functional active wound dressings. Therefore, developing new approaches by loading sEVs into ingenious biomaterials is expected to solve application bottlenecks of injection of free sEVs.

### 5.2 Combination with biomaterials

Short half-time and rapid clearance *in vivo* have been the challenges for injecting free sEVs in practical applications (Golchin et al., 2022). Due to high designability of biomaterials, researchers have combined sEVs with innovatively designed biomaterials to prolong the retention time to increase their utilization, and achieve on-demand release *in vivo* (Las Heras et al., 2020). In this section, we discussed and overviewed some ingenious symphonic designs between sEVs and advanced biomaterials like hydrogel pad, microneedle array, microspheres or electrospun nanofibers.

#### 5.2.1 Hydrogel pad

Hydrogel pad with high biocompatibility has been explored widely to function as delivery system for cells or drugs in the treatment of wound healing. Furthermore, it can offer an appropriate microenvironment to facilitate migration and proliferation of cells at wound site (Peppas et al., 2006). In 2020, Akbari et al. reviewed the application of biocompatible hydrogels for delivering natural and engineered exosomes (Akbari et al., 2020). Here, based on its high adjustability on composition and structure, we overviewed the applications of hydrogel pads with single layer or bi-layer in promoting wound healing process.

To prolong retention time of sEVs, we encapsuled VH298-EVs in 15% gelatin methacryloyl (GelMA) hydrogel which has attracted great attraction in regenerative medicine because of its injectable and UV-crosslinked properties. We compared the *in-vitro* and *in-vivo* release behavior of VH298-EVs from 15% GelMA hydrogel or in free form. For *in-vitro* result, PKH26 labeled VH298-EVs released from 15% GelMA hydrogel could still be detected after co-culturing with human vascular endothelial cells (HUVECs) for 72 h which is longer than that in free form (about 48 h). Additionally, the fluorescence signal of PKH26 used to label VH298-EVs from 15% GelMA distributed evenly around the wounds at day 4 by *in-vivo* imaging system, in comparison with free VH298-EVs with 4-point injection method (Wang et al., 2022). To improve retention time, Ma et al. incorporated engineering sEVs secreted by NR8383 cells stimulated with lipopolysaccharides (LPS) and bioglass (BG) ion extracts (LPS/BG-exos) into a macro-porous hydrogel composed of sodium alginate (SA) and hyaluronic acid (HA) (Ma et al., 2022a). The macro-porous hydrogel was crosslinked with calcium ion and UV consecutively. The release behavior of LPS/BG-exos from macro-porous hydrogel pad was studied by immersing LPS/BG-exo-loaded macro-porous hydrogel pad into 1 mL PBS at 37°C and the concentration of released LPS/BG-exos was measured by BCA Protein Assay Kit. About 50% of LPS/BG-exos showed burst release from macro-porous hydrogel within 6 h and they were released completely at day 8, indicating that the macro-porous hydrogel pad can control the release behavior of LPS/BG-exos to achieve better therapeutic effect. However, the physical adsorption of sEVs in hydrogel pads will result in a burst release of sEVs. To meet this challenge, Fan et al. (2022) encapsuled BMSC-sEVs into a dual-network electroconductive hydrogel composed of GelMA, polypyrene, and tannic acid (TA). The abundant polyphenol groups in TA facilitated the immobilization of BMSC-sEVs. About 80% of BMSC-sEVs were released from hydrogel without TA immediately and retention time was only 7 days. In sharp contrast, the retention time of BMSC-sEVs from hydrogel with TA was evaluated as 14 days, leaving enough time for them to exert their effects.

However, the hydrogel pad with single layer cannot meet the challenge of perfect skin repair, especially scarless wound healing, because the single layer of hydrogel pad cannot realize multi-step release of sEVs. Therefore, a hetero-structured hydrogel pad was developed in which the upper- and lower-layers were loaded with two kinds of sEVs. The different swelling rates between the upper- and lower-layers result in the sequential release of sEVs, performing different functions for promoting wound healing and inhibiting scar formation, respectively (Shen et al., 2021). To our knowledge, MSC-sEVs-loaded hydrogels with three or more layers for faster wound healing have not yet been reported. But we believe that loading sEVs with various functions in different layers of multi-layer hydrogel pads is expected to match each stage of wound healing accurately and achieve perfect skin repair.

#### 5.2.2 Microneedle array

Although hydrogel pads can realize sustained release or multi-step release of sEVs, traditional hydrogel pads cannot pass skin barrier, discounting the therapeutic effect of drugs. As a promising delivery system, microneedle array can transport macromolecules, drugs or sEVs with their microscale needle arrays through skin’s barrier in a minimally invasive manner, realizing long-term delivery of bioactive components (Chen et al., 2021). Yuan et al. encapsuled HUVECs-derived sEVs (H-EVs) and tazarotene into a microneedle patch composed of GelMA and polyethylene glycol diacrylate (PEGDA). *In-vitro* and *in-vivo* experiments demonstrated that this microneedle system realized sustained release of PKH26-labeled H-EVs and reached over 80% cumulative release amount after 10 days (Yuan et al., 2022). In another study, Ma et al. reported a core-shell-structured microneedle array in which ferrum-pretreated MSC-derived artificial nanovesicles (Fe-MSC-NVs) were packed into the inner hyaluronic acid (HA) core and polydopamine nanoparticles (PDA NPs) were encapsuled in the outer methacrylated hyaluronic acid (HAMA) shell. The height of 860 μm allowed this core-shell-structured microneedle array to penetrate the skin for the delivery of Fe-MSC-NVs and PDA NPs. Besides, the core-shell structure allowed PDA NPs and Fe-MSC-NVs to be released at different healing phase to scavenge ROS-mediated inflammation reaction and to accelerate the proliferation, as well as angiogenesis (Ma et al., 2022b). In future study, the spatiotemporal-controlled release of sEVs by the stimuli-responsive microneedle array like physiological signal stimuli (pH, glucose and enzymes) or physical signal stimuli (temperature, light or mechanical stress) used in the treatment of cancer or diabetes (Makvandi et al., 2021) may shed light on the precise regulation of the skin wound healing process.

#### 5.2.3 Microspheres

Although extensive studies have been reported to encapsule sEVs in hydrogels or microneedle arrays, the uniform distribution and release rate of sEVs from those materials are usually uncontrollable and rapid (Chen et al., 2022b). Microspheres produced by microfluidic technology show uniform size and longer release time (release kartogenin for up to 5 weeks) (Yang et al., 2020a), arising great interest in delivering drugs, growth factors, and nanophase materials. Chen et al. fabricated a biofunctional microsphere through microfluidics technology in which Tβ4-sEVs were encapsuled by photocrosslinked GelMA and PEGDA. The continuously released Tβ4-sEVs (until about 21 days) improved the angiogenesis activity of coronary endothelial cells (CAECs) *via* the miR-17-5p/PHD3/Hif-1α pathway (Chen et al., 2022b). Recently, by electrostatic interaction, Cai et al. immobilized hypoxic sEVs (H-sEVs) on PDA-coated injectable porous poly (lactic acid-co-glycolic acid) (PLGA) microspheres, achieving sustained release of H-sEVs until 21 days without decayed bioactivity and promoting vascularized bone regeneration (Gao et al., 2022).

#### 5.2.4 Electrospun nanofibers

In the last 2 decades, electrospun nanofibers have been explored widespread in a variety of biomedical applications owing to their tunable morphologies and biophysical chemistry properties including diameters, patterns, surface modification, and mechanical properties (Xue et al., 2019). In terms of regenerative medicine, the electrospun scaffolds composed of nanofibers showcased similar morphology and modulus to ECM, supporting migration and growth of cells (Xue et al., 2019). By employing electrostatic interaction between electropositive polyethyleneimine (Wu et al., 2018) and electronegative sEVs, Su et al. immobilized MSC-sEVs on the surface of PEI-coated polycaprolactone (PCL) electrospun nanofibers (Su et al., 2021). This bio-system can modulate the response of macrophages and regulatory T cells around skin wounds in mice exquisitely, in which the PCL fibrous scaffold can act as the “recruiter,” while the immobilized MSC-sEVs function as the “trainer” for immune cells respectively (Su et al., 2021). In another similar study, electronegative hADSCs-Exos were tethered to PLGA nanofibrous scaffold decorated with electropositive Mg-gallate metal organic framework (Mg-GA MOF) to accelerate bone regeneration. hADSCs-Exos released slowly from nanofibrous scaffold can stabilize bone growth microenvironment and promote angiogenic activity, while Mg^2+^ induced the osteogenic differentiation of MSCs and GA offers potent antioxidative and anti-inflammatory abilities (Kang et al., 2022).

Therefore, we have reasons to believe that the wonderful concerto between sEVs and advanced biomaterials will play wonderful movement in the treatment of chronic non-healing wound.

## 6 Scalable production and engineering of sEVs

Although sEVs have been recognized as the potential candidates in clinical applications and have been explored widely, the low yield, inadequate therapeutic effect, and targeting efficiency of sEVs are still stumbling blocks of native sEVs for large-scale clinical applications. Very recently, a workshop, named “massivEVs”, organized by the International Society for Extracellular Vesicles (ISEV), “The Extracellular Vesicle Foundry” (evFOUNDRY), and “Extracellular vesicles from a natural source for tailor-made nanomaterials” (VES4US) also expressed serious concern on the large-scale production of sEVs from sources, upstream and downstream technologies, validation, standardization, and regulation (Paolini et al., 2022). Up to now, numerous ingenious strategies have been developed to conquer those bottlenecks, promoting the application of sEVs in clinical practice. In this section, we will focus on how to increase the yield and achieve the engineering of sEVs on demand for wound healing.

### 6.1 Improving the yield of sEVs

Changing culture environments of parent cells has been reported to be beneficial to increase the yield of sEVs. For example, a bioreactor composed of hollow fibers (Watson et al., 2016) can sustain larger numbers (more than 10^9^ per mL) of cells and produce highly concentrated cell culture supernatants, thus leading to mass production of sEVs. Using this bioreactor, the yield of sEVs can be improved about 40 folds than conventional system. Additionally, oxygen environment like oxidative stress (Atienzar-Aroca et al., 2016) or hypoxic (King et al., 2012) stimulus was also reported to be responsible for the yield of sEVs. Sandra Atienzar-Aroca et al. observed that retinal pigment epithelium cells (RPEs) secreted larger amounts of sEVs when they were exposed under proper oxidative stress (achieved by treating cells with 40 or 80 nM ethanol) (Atienzar-Aroca et al., 2016). The yield of sEVs can be enhanced about 2 folds when the concentration of ethanol in the cell culture medium of PREs was 80 nM, in comparison with that without ethanol. In another study, it is reported that exposing tumor cell lines (MCF7, SKBR3, and MDAMB 231) to the moderate (1% O_2_) and severe (.1% O_2_) hypoxia environments significantly increased the number of sEVs present in the conditioned media (King et al., 2012).

Applying external chemical or physical stimulus has been reported to regulate cellular metabolic behavior of parent cells to improve the yield of sEVs. Up to now, several chemical agents have been investigated to affect and improve the secretion of sEVs including BG (Wu et al., 2021), metformin (Liao et al., 2021), cytochalasin B (Pick et al., 2005), monesin (Savina et al., 2003), and serotonin (Glebov et al., 2015) by adding them to the culture medium of parent cells. These reagents affect the production of sEVs through different signaling pathways in parent cells. For example, the ion products of BG can upregulate the expression of neutral sphingomyelinase-2 (nSMase2) and Rab27a to enhance nSMases and Rab GTPases pathways which is essential to regulate the vesicle formation and membrane traffic in human-derived MSCs (Wu et al., 2021). However, metformin was reported to activate autophagy-associated pathway in MSCs to enhance the production of sEVs (Liao et al., 2021). Monesin (a Na^+^/H^+^ exchanger) can induce exchanges in intracellular calcium, thus stimulating sEV release from K562 cells. Similarly, the involvement of cAMP- and Ca^2+^- dependent signaling pathways was reported as the underlying mechanism of serotonin in the regulation of EV secretion from microglia cells (Glebov et al., 2015).

In addition to those chemical or bio-stimulation, applying high frequency acoustic (Ambattu et al., 2020), low level electricity (Fukuta et al., 2020), mechanical forces with the combination of 3D scaffold (Guo et al., 2021), lower pH (Parolini et al., 2009; Ban et al., 2015), and ionizing radiation (Jabbari et al., 2019) can also increase the yield of sEVs from various cells. For instance, Ambattu et al. reported that applying an AC electric field with high frequency acoustic of 10 MHz on U87-MG and A549 cells with 7 cycles over 280 min achieved about 8 to 10 folds enhancement in the production of sEVs (Ambattu et al., 2020). By the treatment of relative lower electric intensity (constant current, .34 mA/cm^2^) for 60 min, the quantity of sEVs from 3T3-Swiss albino cells and B16F1 cells was enhanced by 1.7 and 1.26 folds, respectively (Fukuta et al., 2020). Guo et al. seeded dental pulp stem cells (DPSCs) or MSCs on Fibra-Cel scaffolds to fabricate a 3D bioreactor and explored the effect of mechanical stimulation with flow stimulation on the yield of sEVs. Surprisingly, the yield of sEVs from above 3D bioreactor under .5 mL/min flow stimulation was improved by about 40.7 and 3.4 folds than the 2D and 3D static counterparts, respectively (Guo et al., 2021).

Overexpressing related regulatory proteins involved in sEV biogenesis *via* gene transfection can also ensure high yields of sEV production. STEAP3, syndecan-4, and L-aspartate oxidase were identified as three sEV production boosters that were involved in sEV biogenesis, the formation of multivesicular bodies, or cellular metabolism to yield more sEVs with high quality (Kojima et al., 2018). In another similar study, activating actomyosin was believed to pull the actin-bound MVB toward the cellular plasma membrane to facilitate the secretion of sEVs (Mittelbrunn et al., 2015). Kai O. Böker et al. reported that overexpression of tetraspanin CD9 significantly augmented the amount of sEVs secreted by different human cell lines, like HEK293, HeLa, SH-SY5Y, as well as B and T lymphocytes (Boker et al., 2018). Other methods such as fusing with synthetic lipid (Jhan et al., 2020), changing cell culture parameters or collection frequency of sEVs (Patel et al., 2017) were also developed to achieve higher yield of sEVs. Although many studies have explored how to improve the production of sEVs, standard operations and unified understanding of underlying mechanisms need to be investigated in depth.

### 6.2 Engineering sEVs

One of the main reasons that sEVs have greater advantages over MSC therapy is that it can be customized to promote wound healing process through on-demand engineered approaches on the parent cells or the isolated sEVs summarized in Table 8. Generally, according to processed objects, developed engineering methods can be classified as engineering on parent cells or engineering on the isolated sEVs.

**TABLE 8:** Current methods for engineering sEVs.

Engineering on parent cells	Engineering sites	Components	Ref	Engineering on isolated sEVs	Engineering sites	Components	Ref
Genetic transfection	Membrane	Membrane proteins	Alvarez-Erviti et al. (2011)	Chemical modifications	Membrane	Cationic lipids, nanoparticles	Nakase and Futaki. (2015); Qi et al. (2016); Tian et al. (2018)
EXPLOR	Lumen	Proteins	(Yim et al., 2016; Choi et al., 2020)	Co-incubation	Lumen	Hydrophobic drugs	Pascucci et al. (2014); Wang et al. (2022)
Co-culturing	Lumen	Hydrophobic drugs	Pascucci et al. (2014)	Electroporation	Lumen	Antisense oligonucleotides, Cas9 mRNA, and guide RNAs, siRNAs, short hairpin RNAs	Kamerkar et al. (2017); Usman et al. (2018)
Cellular nanoporation	Lumen	Therapeutic mRNAs, Targeting peptides	Yang et al. (2020b)	Sonication	Lumen	Hydrophobic drugs	Kim et al. (2018)
Ischemic preconditioning	Lumen	MiRNAs	Li et al. (2015)	Extrusion	Lumen	Hydrophobic drugs, Enzymes	Jang et al. (2013); Haney et al. (2015)
Inflammatory cytokines	Lumen	MiRNAs	Domenis et al. (2018)	Saponin-assistant permeabilization	Lumen	Hydrophilic drugs	Fuhrmann et al. (2015)
				Lipid-assistant chemical transfection	Lumen	SiRNAs	Shtam et al. (2013)
				Hydrophobic modification	Lumen	SiRNAs	Didiot et al. (2016)

Up to now, several methods have been developed by researchers to customize the contents of sEVs by operating parent cells. Genetic engineering is recognized as a common strategy to enrich non-coding RNAs and proteins in the lumen or on the surface of sEVs. By virtue of the transfection of hBMSCs by miR-29b-3p lentiviral vector, the miR-29b-3p-enriched sEVs were generated to suppress excessive capillary proliferation and collagen deposition in the late proliferation and remodeling phases of wound healing. By transfecting HEK293 cells with H19-overexpressing (H19-OE) lentiviral vector and followed by a gradient extrusion method, long non-coding RNA (LncRNA)-H19 was encapsuled into sEV-mimetic nanovesicles (Tao et al., 2018). The obtained H19-sEV-mimetic nanovesicles were able to counteract the regeneration-inhibiting effect of hyperglycemia, thus accelerating the healing process of diabatic wounds. Similar method was employed to load SERPINA1, SERPINF2, and SERPING1 into sEVs respectively to promote healthy ECM and facilitate the formation of a beneficial fibrin scaffold and the resolution of the inflammation phase during wound repair (Park et al., 2022). Moderating culture environment of parent cells can also modulate the content or the therapeutic effect of sEVs. The angiogenesis efficacy of MSC-derived sEVs can be improved by applying an ischemic preconditioning on MSCs for 1 h (Li et al., 2015), which might be a useful solution to boost angiogenesis at wound site. Very recently, our colleagues reported TGF-β1-enriched sEVs by pretreating HUMSCs with isoproterenol (ISO) under anoxic environments. In this study, ISO was employed to promote the secretion of TGF-β1, while the hypoxic environment could boost the yield of sEVs. The engineered sEVs enhanced the migratory behavior and stem cell properties of epidermal keratinocytes and accelerated wound re-epithelization *in vivo*. To mimic inflammatory microenvironment, lipopolysaccharide (LPS) was employed to stimulate hUC-dMSCs to generate sEVs enriched with miRNA let-7b. They could mitigate inflammation and accelerate diabetic wound healing by upregulating the expression of anti-inflammatory factors and inducing macrophages to shift to anti-inflammatory M2 phenotype (Ti et al., 2015). Pretreating adipose MSCs with inflammatory cytokines like IFNγ or TNFα would enhance the anti-inflammatory efficacy of adipose MSCs-derived sEVs, shifting macrophages from M1 to M2 phenotype (Domenis et al., 2018), which provided a clue for the management of inflammatory process at wound site.

Moreover, several strategies were also proposed to augment the targeting efficacy or therapeutic efficiency of isolated sEVs for accelerating wound healing. To improve therapeutic efficiency, different kinds of cargoes like non-coding RNA, proteins, and drugs were loaded into sEVs. Co-incubation may be the simplest solution to encapsule cargoes into sEVs (Pascucci et al., 2014). Very recently, our group loaded VH298 (a stabilizer of HIF-1α) into sEVs successfully by co-incubating them at 37°C for 1 h (Wang et al., 2022). The VH298-loaded sEVs facilitated the migration and tube formation of HUVECs by activating HIF-1α pathway, thus promoting angiogenesis during wound healing process. Unlike the soft phospholipid bilayer membrane of their parent cells, the high level of cholesterol and sphingomyelin on the membrane of sEVs makes them rigid and difficult to open at room temperature. Therefore, enhancing the permeability of phospholipid bilayer membrane of isolated sEVs is the prerequisite to regulate the contents of isolated sEVs. It has been reported that aggressive environments are needed to destroy membrane structures of sEVs and promote drug fusion. In general, the permeability of phospholipid bilayer membranes can be increased by electroporation (Kamerkar et al., 2017; Usman et al., 2018; Lv et al., 2020), freeze-thaw (Gondaliya et al., 2022) and so on. A modified calcium chloride (CaCl_2_) transfection was used to load miR-155 inhibitor into the lumen of BM-MSC-sEVs by letting the mixture of miR-155 inhibitor, sEVs, and CaCl_2_, as well as PBS go through a freeze-thaw cycle. The synergistic effects between miR-155 inhibitor and sEVs facilitated the migration of keratinocytes and rebalanced FGF-7 and MMPs levels, thereby accelerating wound healing (Gondaliya et al., 2022). In another study, electroporation was employed to encapsule miR-21-5p mimics into adipose MSC-sEVs. The engineered sEVs enhanced the proliferation and migration behaviors of keratinocytes by activating Wnt/β-catenin pathway and then facilitated diabetic wound healing *in vivo* (Lv et al., 2020). The great progress in increasing the yield of sEVs and in engineering sEVs will further promote the clinical applications of sEVs in chronic non-healing wounds.

## Conclusion and prospects

As summarized here, findings from various investigations have implied that extensively sourced MSC-sEVs hold great therapeutic potency for treating chronic non-healing wounds (Lou et al., 2021). The curative benefits can be achieved by functional components in MSC-sEVs from the following main aspects. Firstly, MSC-sEVs could restrict overwhelmed inflammation by modulating immune cell portions and their behaviors and re-balancing cytokines toward an anti-inflammatory role in inflammatory wounds. Secondly, these MSC-sEVs can stimulate angiogenesis which guarantees adequate oxygen and nutrition supply to wound sites. Thirdly, they can also manage to suppress senescence-associated secretory phenotype (SASP) to rejuvenate skin cells and to achieve favorable healing process. Fourthly, re-epithelialization is significantly enhanced after MSC-sEV employment. Finally, application of MSC-sEVs can help to amend scarring and achieve a regenerative repair by priming fibroblasts and optimizing collagen distribution (Li et al., 2022).

Non-etheless, a series of concerns and flaws should be overcome before we bring MSC-sEVs into clinical setting. Firstly, MSCs derived from multiple sources and cultured under disunited conditions will release sEVs with diverse components. As a result, controversies arise about reliable functionality and reproducible results. Next, for now, there are no agreed or existing uniform criterions for isolation, identification or qualitative methods, lacking of industry standardization. In addition, native MSC-sEVs are sophisticated compartments and contain various molecules, such as RNAs, proteins, and chemokines. Hence, it’s hard to determine the most effective constituent, impeding further MSC-sEVs clinical application. Another obstacle is the fact that the yield and efficiency of native or unmodified MSC-sEVs haven’t yet been satisfactory. Although researchers have found ways to improve productivity, functionality, and targeting efficacy, how to develop a large-scale standardized method to enhance the throughput and therapeutic efficiency of MSC-sEVs is still a major constraint that limits clinical application of MSC-sEVs in the future. Additionally, the operating methods on the cellular behavior of parent cells or the effect on other content inside of sEVs needs further exploration. Finally, agreed requirements for producing and quality control should be a must to guarantee the safety and potency of MSC-sEVs. A bulk of data about MSC-sEV employment has accomplished from *in-vivo* preclinical experiments which may not inexorably mirror the clinical characteristics. Thus, further research is still imperative to unveil the advantages and disadvantages of clinical application of MSC-sEVs on chronic non-healing wounds. After above issues being resolved, it is believed that MSC-sEV therapy will be a potential and encouraging method for rapid and complete regeneration of chronic non-healing wounds.
